# Accuracy of Digital Orthodontic Treatment Planning: Assessing Aligner-Directed Tooth Movements and Exploring Inherent Intramaxillary Side Effects

**DOI:** 10.3390/jcm13082298

**Published:** 2024-04-16

**Authors:** Ludger Keilig, Anna Fittgen, Helen Schneider, Rafet Sifa, Jörg Schwarze, Christoph Bourauel, Anna Konermann

**Affiliations:** 1 Oral Technology, University Hospital Bonn, 53111 Bonn, Germany; 2 Department of Prosthodontics, University Hospital Bonn, 53111 Bonn, Germany; 3 Department of Orthodontics, University Hospital Bonn, 53111 Bonn, Germany; 4 Fraunhofer-Institute for Intelligent Analysis- and Informationsystems IAIS, 53757 Sankt Augustin, Germany; 5 Bonn-Aachen International Center for Information Technology (B-IT), LAMARR Institute for Machine Learning and Artificial Intelligence, University of Bonn, 53115 Bonn, Germany; 6 Private Practice, 50674 Cologne, Germany

**Keywords:** aligner, digital treatment planning, orthodontics, side effects, treatment accuracy

## Abstract

**Background:** The attainment of precise posterior occlusion alignment necessitates a deeper understanding of the clinical efficacy of aligner therapy. This study aims to determine whether the treatment goals defined in the virtual planning of aligner therapy are effectively implemented in clinical practice, with a particular focus on the influence of distalization distances on potential vertical side effects. **Methods:** In this retrospective, non-interventional investigation, a cohort of 20 individuals undergoing Invisalign^®^ treatment was examined. Pre- and post-treatment maxillary clinical and ClinCheck^®^ casts were superimposed utilizing a surface–surface matching algorithm on palatal folds, median palatine raphe, and unmoved teeth as the stable references. The effectivity of planned versus clinical movements was evaluated. Groupings were based on distalization distances, planned vertical movements, and Class II elastic prescription. Statistics were performed with a two-sample t-test and *p*-value < 0.05. **Results:** Clinically achieved distalization was significantly lower than virtually planned distalization, regardless of additional vertical movements, where a lack of implementation was contingent upon the extent of distalization, with no mitigating effects observed with the application of Class II elastics. Intriguingly, no adverse vertical side effects were noted; however, the intended intrusions or extrusions, as per the therapeutic plans, remained unattainable regardless of the magnitude of distalization. **Conclusions:** These findings underscore the imperative for future investigations to delve deeper into the intricacies surrounding translational mesio-distal and vertical movements, thereby enhancing predictability within orthodontic practice. To facilitate successful clinical implementation of vertical and translational movements via aligners, the incorporation of sliders emerges as a promising strategy for bolstering anchorage reinforcement.

## 1. Introduction

In orthodontics, tooth movement can be affected by different removable and fixed appliances that are utilized according to the patients’ treatment needs. The clear aligner technique has become increasingly popular for patients over the years, especially because of their aesthetic advantages [1].

Clear aligner treatment (CAT) is expected to gain more importance not only in the clinical field but also in academic research, as evidenced by progressing research efforts and citations, contributing to a broadened knowledge of its potential benefits and challenges [2]. In view of increasing experience in the clinical use of aligners, improvement in the systems, and the optimization of aligner materials, the range of indications for CAT has continued to expand and has become even more predictable in terms of treatment results for patients [3]. As a result, complex cases involving extraction, extrusion, or distalization can be effectively managed based on the literature findings, with treatment outcomes comparable to those achieved with multi-bracket appliances as the gold standard [4,5]. The growing demand for inconspicuous treatment methods further drives aligner research, and the clinical experience of practitioners in the field of digital orthodontics is also growing continuously. It can therefore be assumed that aligner treatments will continue to gain importance in the future, which also underlines the need for further research in this area [6].

In addition to all of these advantages and progress, aligner therapy confronts practitioners with new challenges regarding possible side effects of this treatment approach, underlined by publications revealing system-inherent drawbacks and limitations of CAT. A systematic review assessed the effectiveness and efficiency of clear aligners versus fixed appliances in the treatment of complex orthodontic cases with premolar extraction, finding shorter treatment durations with fixed appliances alongside superior buccolingual inclination and occlusal contact management, though noting potential disparities in predicted versus achieved tooth movements with CAT [7]. Another systematic review analyzed the effectiveness and predictability of CAT by comparing treatment outcomes with fixed appliance therapy, identifying a low-to-moderate level of certainty in the efficiency of specific tooth movements, suggesting that clear aligners may achieve clinically acceptable results for mild-to-moderate malocclusions, whereby many movements may still lack predictability despite recent system improvements [8].

Recent studies have revealed that occlusal contacts, in particular, are a problematic issue as they are difficult to adjust via CAT, showing a decrease in posterior occlusal contact in Class I mild-to-moderate malocclusion upon treatment; this was associated with deficiencies in achieved buccolingual inclination and transverse expansion of the posterior teeth, as planned bodily expansion proved to be ineffective, with most expansion occurring through unplanned buccal tipping [9,10]. These results are supported by other works, assuming that these side effects could be caused by the complete encasement of teeth by the material [11,12]. An investigation on maxillary expansion or contraction and adjustment of occlusal contacts found that the effectiveness in achieving these virtually planned transversal targets was 45% and concluded that orthodontists should consciously use overcorrection for transversal maxillary expansion or contraction to compensate for the lack of clinical implementation [13] Another study analyzed the efficiency of Invisalign^®^ aligners for the correction of crowding in the anterior region by superimposing initial and final models on unmoved, stable teeth in the posterior region [14]. Here, the second molars were ultimately excluded for superimposition from the study design, as no satisfactory congruence with the initial model could be achieved with them. This suggests that wearing aligners can already lead to changes in tooth position as a side effect, even if no movement is planned for those teeth. Another study also found that the thickness of the aligner material can lead to unwanted intrusion of the posterior teeth, but so far there are a lack of studies that accurately measure intrusion and analyze its causes [15]. Two other studies have looked at intrusions and extrusions as side effects of the resulting opposing forces, and both equally conclude that vertical posterior movements having an influence on overbite occur mainly in the molar region of the mandible [16,17]. Another study revealed that vertical movements are the least effective movements to implement alongside derotation of round teeth, but without quantifying this precisely in its investigations [18]. Although the adjustment of overbite and posterior occlusion are key criteria for successful orthodontic treatment, a quantification and analysis of the vertical side effects in the posterior region during aligner treatment have been lacking to date.

In order to be able to implement difficult movements in a clinically satisfactory manner, despite these weaknesses in aligner treatment, it is now common practice for many practitioners, due to their increasing level of knowledge, to adapt the planning so that certain movements are overcorrected [19,20]. Furthermore, aids such as pressure point pliers, attachments, buttons, and elastics are also becoming increasingly widespread to support movements and improve treatment outcomes [21]. Nevertheless, it would be desirable to be able to carry out aligner treatment without having to take measures aimed solely at avoiding side effects. Systematic research into aligner-specific side effects, their quantification, and the analysis of the corresponding causes, to which we would like to contribute with our work, is the basis for improving the system in this respect.

The aim of our study was to analyze system-immanent, intramaxillary side effects of aligner therapy during distalization for tooth position correction. The hypotheses of our study were as follows.

First, our null hypothesis was that planned and achieved distalization are equal, regardless of whether additional intrusion or extrusion or only translational distal movement is planned.

Second, we hypothesized a correlation between the extent of distalization and the extent of mesio-distal side effects in the sense that the greater the distance of planned distalization, the less clinically achievable it is.

Third, we expect that wearing Class II elastics will positively enhance the efficiency of distalization.

Fourth, we assume that vertical side effects occur during distalization, where we expect intrusion rather than extrusion due to the splint and masticatory forces.

Finally, we postulate a correlation between the extent of distalization and the extent of vertical side effects, in the sense that the greater the distance of planned distalization, the more vertical side effects occur.

To achieve greater precision in aligning posterior occlusion in clinical practice, this study utilizes computer-aided superimpositions via a surface–surface matching algorithm to test our hypotheses regarding the clinical behavior of teeth compared to virtually planned movements.

## 2. Materials and Methods

### 2.1. Patient Collective and Selection Criteria

The non-interventional retrospective study was conducted in accordance with the Declaration of Helsinki. The patients had already finalized their treatment, which was conducted according to their orthodontic indication, before the beginning of this study and had given their written consent to participate in the treatment in advance.

The patient collective examined in this study was determined as follows. All cases exclusively treated by the same practitioner with over 20 years of experience with the Invisalign^®^ system between 2014 and 2020 were screened. Inclusion criteria were bilateral distalization in the maxilla with a protocol of sequential distalization of molars and semi-sequential distalization of premolars, presence of second molars, an age of at least 25 years for exclusion of side effects due to residual growth, no wisdom teeth with an extraction date at least 1 year before the start of treatment, no previous orthodontic treatment in the last 2 years before the start of treatment, and periodontal health. Exclusion criteria were lack of compliance, medication impairing bone metabolism, periodontal problems, and mid-course corrections. The planned protocol for aligner use provided for a wearing time of 22 h/day and a change mode between 4 and 9 days depending on the individual’s reaction. Patient compliance was accounted for by excluding all patients with an aligner change mode greater than 10 days. Furthermore, the wearing time of the aligners per day was queried at each appointment and the current aligner number was noted to determine insufficient wearing time during treatment. Based on these selection criteria, a total of 73 cases were identified as being potentially eligible. After ensuring the completeness of documentation, the number was reduced to 48 cases. Subsequently, during quality control of the impressions to assess integrity and suitability for palatal fold overlay, an additional 28 cases had to be excluded, resulting in 20 cases being deemed suitable for this study.

The mean age of the selected patients at baseline was 40.2 ± 10.1 years. The group comprised 15 women and 5 men. The average interval between aligner changes was 6.6 ± 1.7 days, and an average of 84.5 ± 11.9 aligners were worn per patient. The average duration of treatment was 82.6 ± 26.6 weeks. All patients were treated with Invisalign^®^ aligners made of SmartTrack™ material with a layer thickness of 0.75 mm. The attachment geometries were customized according to the planned tooth movements, whereby the 2nd molars received no attachments throughout. Since 16 patients also received distalization in the mandible, the use of Class II elastics (3/16, 3.5 Oz) to reinforce anchorage in the maxilla was planned individually, with the Class II elastics being worn every night in 11 patients and every other night in 5 patients.

### 2.2. Formation of Groups

The categorization presented below was conducted independently for each patient’s individual teeth. Consequently, patients were typically divided into multiple groups based on the planned movements for their teeth.

The initial step of treatment for the entire patient cohort involved the distalization of second molars. The first categorization was determined by the extent of distalization, resulting in groups D1 for slight distalization of 0–1 mm, D2 for moderate distalization of 1.1–2 mm, and D3 for pronounced distalization exceeding 2 mm, as outlined in Table 1.

Subsequently, the second categorization focused on planned vertical movements. Teeth either lacking vertical movement plans or with movement less than twice the anticipated measurement error of 0.1 mm (i.e., 0.2 mm) were placed in group V0. Teeth with extrusion exceeding 0.2 mm were designated as group Ve, while those with intrusion beyond 0.2 mm were categorized into group Vi.

Lastly, the third categorization was determined by the prescription of Class II elastics. Patients were stratified into group E0 if no elastics were prescribed, group E1 if elastics were applied every other night, and group E2 if elastics were applied nightly.

### 2.3. Data Preparation Process

Four different models were used per patient for the superimpositions. These included the two clinical models (C_i_, initial model, and C_f_, final model) and the virtual Clincheck^®^ models (V_i_, initial model, and V_f_, final model). The clinical models were digitized with an iTero^®^ Element 1 scanner and converted into STL files. The ClinCheck^®^ models were exported from the software directly as STL files. Since the basis for virtual treatment planning using ClinCheck^®^ is a scan of the patient’s dentition at the start of treatment (V_i_), which was also created via iTero^®^ Element 1 intraoral scanner, C_i_ and V_i_ should be congruent. V_f_ shows the desired treatment goal, whereby C_f_ as a clinical outcome should ideally also be congruent with this, if all movements have been implemented 100%, or deviate from this if side effects occur.

### 2.4. Alignment of the Models for Superimpositions

**Figure 1:**
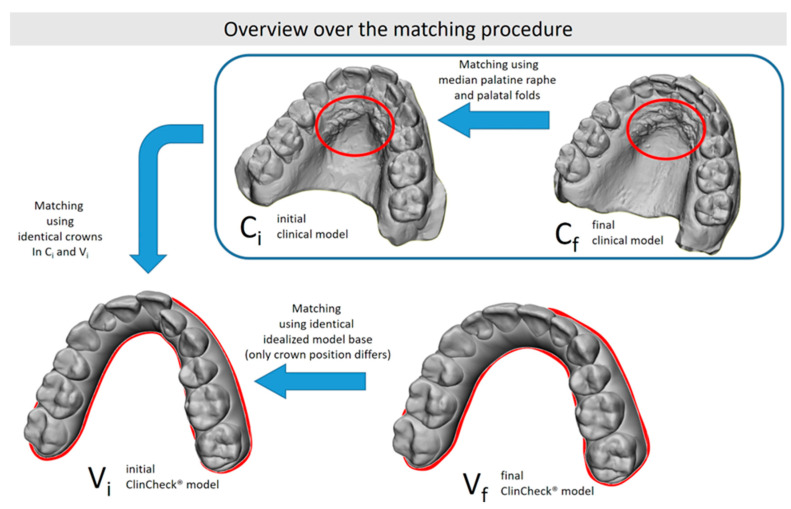
Alignment of the different models using shared features between different model pairs: the initial clinical and ClinCheck^®^ models share the same crown positions, while both clinical models share the same palatal folds (marked in red).

In order to determine crown movements between the different models, all models had to be placed into the same reference system necessitating alignment to a common global coordinate system. The procedure of this alignment is schematically shown in Figure 1. The whole procedure was carried out in 3D graphics software (Surfacer 10.5, Imageware/Siemens PLM Software, Plano, TX, USA).

Alignment was performed as follows: given the standard pre-processing of virtual models by Invisalign^®^ to ensure compatibility with ClinCheck^®^ software, the ClinCheck^®^ models were oriented in such a way that the occlusal plane was parallel to the *x*/*y* plane, with the midline pointing to the *y*-axis. We therefore used the initial ClinCheck^®^ model (V_i_) as the reference for all alignments. The final ClinCheck^®^ model (V_f_) was already in the same reference system, as it was generated based on the V_i_ model.

Since both models V_i_ and C_i_ must be identical due to production before active treatment without any tooth movement being conducted, the teeth of C_i_ and V_i_ could be aligned via a surface–surface matching algorithm [22], overlaying the unmoved teeth of the initial situation and the ClinCheck^®^, respectively, according to the principle of distance minimizing.

**Figure 2:**
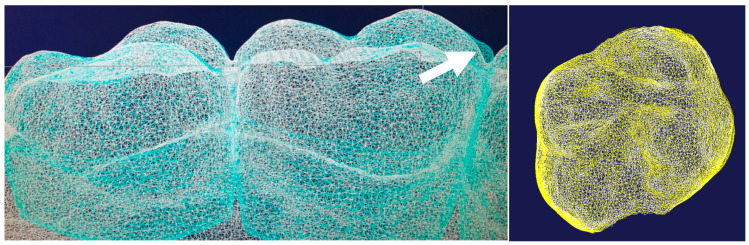
The left-sided figure presents a satisfactory overlay of the C_i_ (cyan) and V_i_ (white) models in wireframe view. However, the discrepancy at the mesial cusp of the first molar (arrow) has to be noted, which is probably due to an impression error. The right-hand figure shows a perfect superimposition of tooth 26, visible on matching yellow and white nodes with congruent contours from every angle.

As the last step, the aligned model C_i_ and the, at this point, unprocessed model of C_f_ were then aligned to each other using the anterior palate and the median palatine raphe as key anatomical landmarks. As a midline correction involves changes in the papilla incisiva and the first two pairs of palatal folds, these structures were selectively removed in case of midline correction to ensure optimal overlap with the stable remaining palatal structures. Alignment utilizing the median palatine raphe and the palatal folds of the anterior palate was chosen, as these structures represent stable and reliable anatomical reference points for studies on tooth movements, with reported accuracies of 0.2 mm for translations and 1° for rotations [22,23]. An exemplary procedure is presented in Figure 2.

### 2.5. Measurement of Individual Tooth Movements

Following alignment of the models, tooth crowns were segmented into separate surfaces and the segmented crown from the initial model was matched to its counterpart in the corresponding final model to determine the movement. As the sharp edges introduced during this segmentation might influence the quality of the matching, special attention was taken to segment corresponding crowns in one model series identically. A visual inspection was performed to assess matching accuracy for each crown and in case of discrepancies, adjustments were made to the segmentation and matching process until accurate superimposition was achieved.

The matching resulted in three translations along the global *x*-, *y*-, and *z*-axis (Tx, Ty, Tz) and three rotations around the global *x*-, *y*-, and *z*-axis (Rx, Ry, Rz).

**Figure 3:**
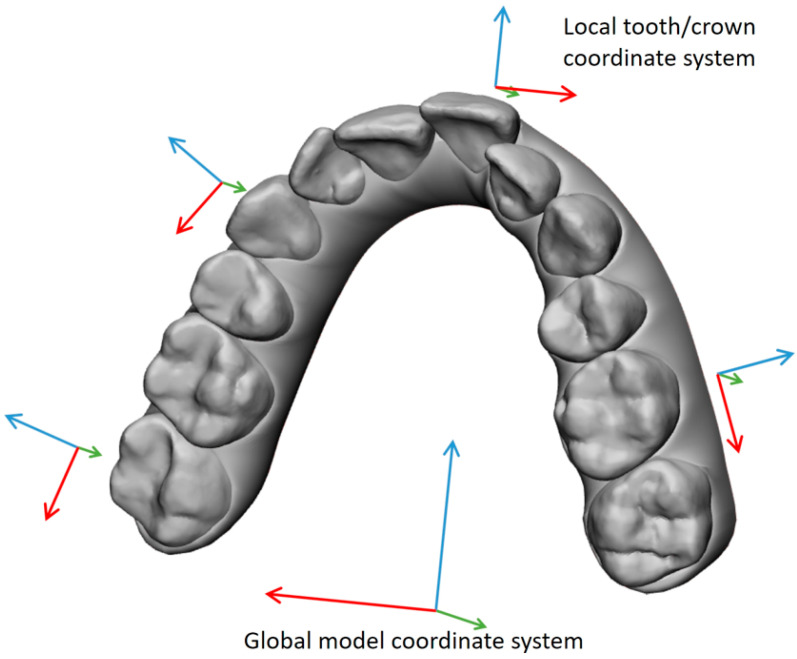
Local coordinate systems were created for each crown in the dental arch based on the initial ClinCheck^®^ model, V_i_. The local coordinate systems allow one to describe the determined tooth movement using the correct dental terminology, like ‘mesio-distal’ instead of ‘along the *x*-axis’.

To describe all determined tooth movements in a manner applicable to clinical practice, a distinct local coordinate system was established for each crown within every patient, referencing the corresponding crown surface in the V_i_ model (Figure 3). The local *x*-axis was assigned to the transversal, the local *y*-axis was assigned to the sagittal, and the *z*-axis was assigned to the vertical direction. Instead of the longitudinal tooth axis, which cannot be determined via ClinCheck^®^, the global *z*-axis from the coordinate system was used, which was defined by a plane corresponding to the occlusal plane.

The local crown coordinate system originated from the Vi crowns by determining the midpoint of the minimum and maximum *x* and *y* coordinates and the maximum *z*-coordinate within the crown point set. Subsequently, a coordinate transformation was applied to convert the determined global movement of the crown surfaces into clinically applicable local tooth movements.

### 2.6. Data Analysis and Statistics

The evaluation of planned versus clinically realized movement for each tooth included assessing measurement accuracy by repeatedly analyzing data from a randomly selected patient at five regular intervals throughout the data collection period to account for potential practice-related improvements leading to more precise evaluations over time.

#### Statistics Were Performed for the Following Hypotheses

1. Whether planned and clinically achieved distalization are equal, regardless of whether additional intrusion or extrusion or exclusively translational distal movement is planned.
2. Whether there is a correlation between the extent of distalization and the lack of feasibility to achieve the final tooth position.
3. Whether wearing Class II elastics positively enhances distalization efficiency.
4. Whether vertical side effects occur during distalization.
5. Whether the extent of distalization and the extent of vertical side effects are correlated.

Statistical analyses were performed using a two-sample *t*-test for independent samples to evaluate the statistical significance of differences between groups. The significance level was set at α = 0.05 with a *p*-value < 0.05.

## 3. Results

This study hypothesized that planned and clinically achieved distalization would be equivalent across different treatment groups regardless of additionally planned vertical movements, and found statistically significant differences between planned and achieved distalization in 96 samples for group V0, 61 samples for group Vi, and 101 samples for group Ve, without distinguishing between different distances of distalization at this point.

**Figure 4:**
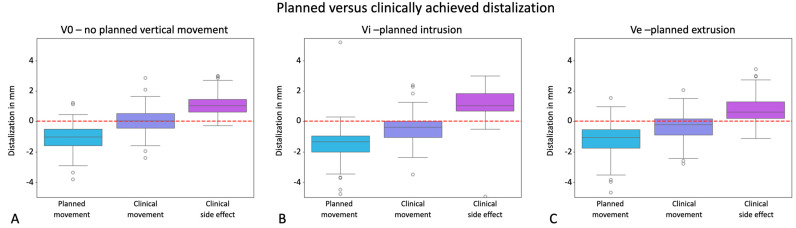
Effectiveness of distalization visualized in the y-axes of the graphs in mm for the groups V0 = exclusive distalization (**A**), Vi = distalization plus planned intrusion (**B**), and Ve = distalization plus planned extrusion (**C**). Values with a positive sign stand for mesial movements; values with a negative sign stand for distal movements. The boxplots visualize the planned movement, the clinically implemented movement, and the deviation resulting from these two boxplots as a side effect in a third boxplot. Dots represent outliers. The red line marks zero translational movement.

Analyses revealed statistically significant differences (*p* < 0.001) between the planned and achieved distalization movements in all of the groups analyzed. The boxplots in Figure 4 clearly illustrate that the distalization movements, depicted by negative values on the *y*-axis, fell significantly short of the planned values, particularly evident in V0 where they almost approached zero. Consequently, our null hypothesis was rejected.

**Figure 5:**
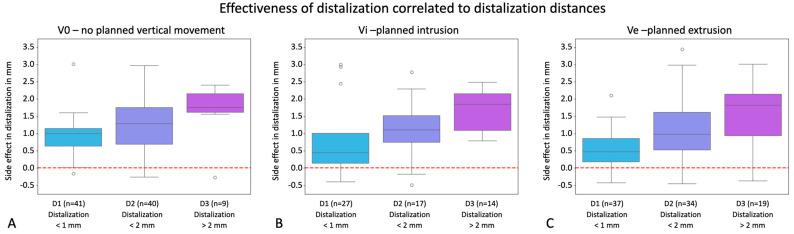
Effectiveness of distalization correlated with distalization distances visualized in the y-axes of the graphs in mm for the groups V0 = exclusive distalization (**A**), Vi = distalization plus planned intrusion (**B**), and Ve = distalization plus planned extrusion (**C**). Values with a positive sign stand for mesial movements; values with a negative sign stand for distal movements. The boxplots show the clinical side effects resulting from the discrepancies between planned and clinically achieved movements. Dots represent outliers. Boxplots exhibit the side effects for the groupings D1 (<1 mm distalization), D2 (>1–2 mm distalization), and D3 (>2 mm distalization). In the right-hand bar charts, the number of individual teeth is visualized. The red line marks zero translational movement.

Our second hypothesis proposed a correlation between distalization magnitude and occurrence of side effects, indicating that increased planned distalization would result in decreased clinical attainment. To address vertical movement components, we conducted separate analyses for groups V0, Vi, and Ve, comparing planned versus clinically realized distalization within each group (D1: no to very slight distalization, D2: moderate distalization, D3: marked distalization). Figure 5 depicts boxplots illustrating the clinical side effects of V0, Vi, and Ve for each distalization group, revealing statistically significant differences among groups in most comparisons, with the degree of distalization implementation varying notably based on distalization distance, particularly evident when additional extrusion or no vertical movement was intended. Here, a significantly poorer implementation was observed even with a planned distalization of 1 mm and a significantly worse conversion of movement was observed with distances > 2 mm when additionally planned intrusion was involved.

The corresponding boxplots are visualized in Figure 5. For V0, a t-test between D1 and D2 resulted in a *p*-value = 0.043 with statistical significance, between D2 and D3 it was 0.127, suggesting no statistical significance, and between D3 and D1 it was 0.001, implying statistical significance between these groups. For Vi, a t-test between D1 and D2 exhibited a *p*-value = 0.376 with no statistical significance; it was *p* = 0.021 between D2 and D3, suggesting statistical significance, and 0.022 between D3 and D1, with statistical significance between these groups.

**Figure 6:**
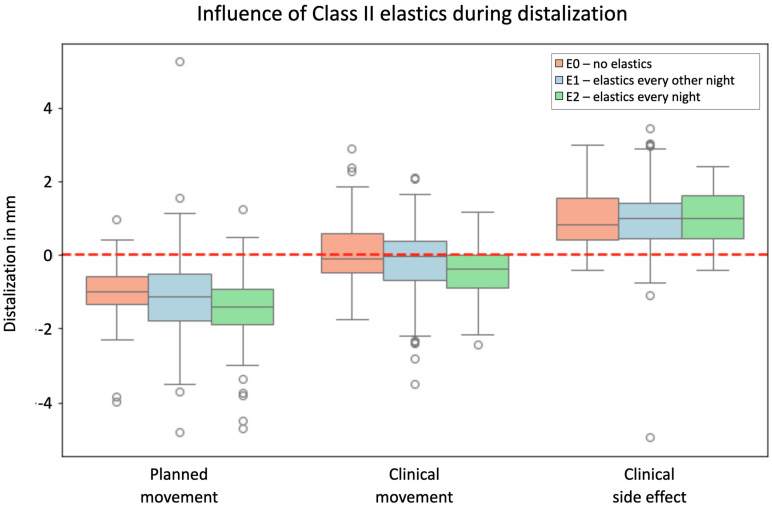
Effectiveness of distalization in relation to wearing elastics, visualized in the *y*-axis of the graph in mm for the groups E0 = no elastics, E1 = elastics every other night, and E2 = elastics every night. Values with a positive sign stand for mesial movements; values with a negative sign stand for distal movements. The boxplots show the planned movement, the clinically implemented movement, and the deviation resulting from these two boxplots as a side effect in a third boxplot shown together for all groupings. Dots represent outliers. The red line marks zero translational movement.

The third hypothesis suggested that wearing Class II elastics enhances distalization efficiency, with statistically significant differences observed in planned versus clinical movement for each group: E0 (no elastics), E1 (elastics every other night), and E2 (elastics every night). The *p*-values of <0.001 for E0, E1, and E2 indicate a significant difference between planned and clinical movement for distalization regardless of elastic application, rejecting the null hypothesis that Class II elastics might positively impact distalization efficiency, with clinical movements being consistently lower than planned movements across all groups, further supported by no significant differences in clinical side effects among groups, as shown in Figure 6.

**Figure 7:**
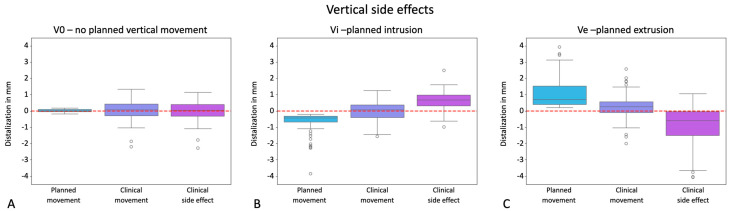
Potential for unwanted vertical side effects occurring during distalization, visualized in the y-axes of the graphs in mm for the groups V0 = exclusive distalization (**A**), Vi = distalization plus planned intrusion (**B**), and Ve = distalization plus planned extrusion (**C**). Values with a positive sign stand for extrusion; values with a negative sign stand for intrusion. The boxplots visualize the planned movement, the clinically implemented movement, and the deviation resulting from these two boxplots as a side effect in a third boxplot. Dots represent outliers. The red line marks zero vertical movement.

Next, we hypothesized that vertical side effects occur during distalization, which was again independently determined for groups V0, Vi, and Ve. No distinctions were made between distalization distance groups at this point. The results presented in Figure 7 revealed no statistically significant difference between planned and clinical movements regarding unwanted vertical side effects for V0 (*p*-value: 0.706). Consequently, if only distalization was planned, this was sufficiently implemented and the final tooth positions could be achieved clinically without unwanted intrusion or extrusion. Statistical analysis indicated significant discrepancies (*p* < 0.001 for Vi and Ve) between planned and clinically achieved vertical tooth movements, with both planned intrusions and extrusions being unattainable, suggesting compromised implementation of vertical prescriptions in treatment planning.

**Figure 8:**
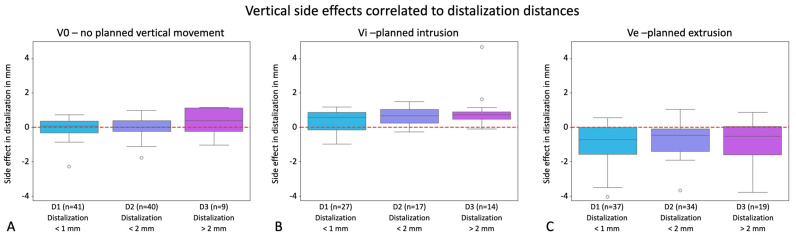
Potential for unwanted vertical side effects occurring during distalization correlated with distalization distances visualized in the y-axes of the graphs in mm for the groups V0 = exclusive distalization (**A**), Vi = distalization plus planned intrusion (**B**), and Ve = distalization plus planned extrusion (**C**). Values with a positive sign stand for extrusion; values with a negative sign stand for intrusion. The boxplots show the clinical side effects resulting from the discrepancies between planned and clinically achieved movements. Dots represent outliers. Boxplots exhibit the side effects for the groupings D1 (<1 mm distalization), D2 (>1–2 mm distalization), and D3 (>2 mm distalization). In the right-hand bar charts, the number of individual teeth in each group is visualized. The red line marks zero vertical movement.

Furthermore, we expected a relationship between the extent of distalization and the extent of adverse events in terms of unwanted intrusion or extrusion. Analyses for each of the groupings were conducted by comparing the groups D1, D2, and D3 regarding vertical side effects. Figure 8 exhibits the boxplots for V0, Vi, and Ve, presenting the clinical side effects for each distalization group to be compared with each other. The bar charts besides the respective graphs visualize the number of teeth in each group. The analyses showed no statistically significant differences between groups D1, D2, and D3, nor for the exclusive distalization group or the ones with additional vertical movement. The calculated p-values were 0.252 (V0, D1-D2), 0.267 (V0, D2-D3), 0.801 (V0, D3-D1), 0.160 (Vi, D1-D2), 0.173 (Vi, D2-D3), 1.809 (Vi, D3-D1), 0.221 (Ve, D1-D2), 0.680 (Ve, D2-D3), and 0.570 (Ve, D3-D1). To conclude, these results imply that potential vertical side effects cannot be correlated with distalization distances and moreover substantiate previous results that vertical side effects do not occur during distalization but are also not implemented clinically if embedded in treatment planning.

## 4. Discussion

The system-inherent initial treatment planning and visualization with the ClinCheck^®^ software in aligner therapy allows the clinical treatment result to be compared with its clearly defined ClinCheck^®^-propagated final outcome, which is not feasible for treatments with a multi-bracket appliance.

The results of the present study show very clearly that the clinically achieved distalization movements were much less than virtually planned, regardless of whether additional vertical movements were to be implemented. The extent of the lack of implementation of distalization was clearly dependent on the distalization distance, whereby this observation was again observed equally for all groups. However, the significance was most pronounced when an additional extrusion or no vertical movement was planned. Here, a significantly poorer implementation was already observed with a planned distalization of 1 mm, but with an additionally planned intrusion from a distance of >2 mm to be covered. In comparison to our results, a study that also investigated the effectiveness of molar distalization using Invisalign^®^ by applying a comparable clinical implementation protocol and also analyzing the data using a superimposition technique showed a very good implementation of these movements [24]. The authors demonstrated an effectiveness of 88.4–86.9% of molar distalization with planned movements > 1.5 mm in the maxilla, which could not be substantiated in this study. These diverse outcomes could be due to the different superimposition strategies of the models, as even though the same superimposition method and the same processing program (Surfacer 10.5) were applied as in our study, they did not utilize the palatal folds and the median palatine raphe as fixed overlay points, but instead utilized unmoved teeth to superimpose the models. In addition, the final model used in this study was taken after treatment was finished, while the comparative study used an intermediate model during the mid-course of therapy in which not all teeth had been moved yet. It can be assumed that the loss of anchorage of the molars occurs primarily during the subsequent distalization of premolars and canines, and accordingly for premolars during canine retraction, which in the other study had not yet or only initially taken place at the time of investigation, implying that the full extent of distalization was not finalized.

Our results moreover revealed that wearing Class II elastics did not positively influence the consistently poor efficiency of the aligners with regard to distalization.

Contrary to the observations in the present study, a study on the distalization of molars with aligners using superimposed lateral cephalometric images before and after the completion of treatment clinically observed a distal movement of 2.25 mm for the first molars and 2.52 mm for the second molars, with all patients wearing 20 h/d Class II elastics to reinforce anchorage [25].

Our investigations also revealed that vertical movements are generally not achieved, with both extrusions and intrusions being undercorrected. However, it appears that wearing aligners does not lead to unintended vertical side effects during distalization, as evidenced by the absence of clinically undesirable intrusions or extrusions.

Moreover, even virtually planned intrusions or extrusions could not be clinically executed as intended, as the system failed to enact these movements irrespective of the distalization distance. The vertical adjustment of posterior teeth using aligners has already been assessed as being problematic in other studies [26,27,28]. With regard to the extrusion of posterior teeth, conceivable causes for unwanted vertical side effects could be the impact of chewing forces and a horizontal growth type, whose mutual interference has already been proven [29], and thus, a differentiation in the effectiveness of vertical movements against the background of the growth type could provide further insights in future studies. A deep bite, as a frequently occurring complex malocclusion with a high risk of recurrence, is associated with changes in chewing and masticatory muscle activity, which must be taken into account during orthodontic treatment as craniofacial characteristics that are detrimental to therapy [30]. In order to successfully implement vertical position changes, consideration should possibly be given to alternatives or, for example, a combination of mini-implants and aligners, which has already been postulated as promising in a study [21]. These alternative treatment combinations could also improve translational distalization over longer distances by reinforcing anchorage in order to be clinically realized [31].

The present study certainly has some methodological weaknesses that have to be discussed. Of mention is that there were differences regarding the Class II elastic treatment regime in the already-small cohort of this study, and there would be further differences depending on the wearing time, which could be one of the reasons for a certain scattering of the data. It should also be mentioned that the superimposition technique might have been compromised due to certain unavoidable preparatory steps. Each time an impression is taken, errors can occur that affect the accuracy of the subsequent plaster cast, for example if the impression is stored for too long. Furthermore, alginates usually shrink to a certain extent, which should be compensated for by the expansion of the plaster after pouring but cannot be verified exactly. Additionally, all plaster casts were digitized using an intraoral scanner and it is unclear whether further inaccuracies occurred in the digital model as a result of this digitalization process.

In summary, the results of this retrospective study on the aligner efficiency of translational distalization movements and potential vertical side effects have both refuted and confirmed our hypotheses as follows.

Distalization is clinically significantly less pronounced than virtually planned. In addition, there is definitely a correlation between the extent of distalization and the extent of side effects, but contrary to our expectations, not with regard to vertical unwanted effects, but with regard to the loss of anchorage in the teeth to be distalized. Wearing Class II elastics does not seem to positively influence these restraints. Vertical undesirable side effects do not occur when distalizing posterior teeth, but intrusions and extrusions are also not implemented if they are virtually planned.

Our results offer a clinical contribution to the improved planning and successful assessment of aligner therapy, taking into account the planned distalization distance. Based on these data, a more targeted use of this appliance is recommended with regard to patient groups with particular reluctance in patients with the need for vertical corrections. Furthermore, additional aids and appliances, such as elastics and sliders, should be taken into consideration to ensure successful treatment results.

## 5. Conclusions

In modern orthodontic treatment, precise virtual planning is essential in ensuring accurate tooth movement with aligners. This study determined the precision of virtually planned posterior distalization in terms of its clinical implementation with CAT while investigating associated potential intramaxillary side effects. As our results clearly showed inadequate clinical realization of movements along with considerable unwanted side effects, it is recommended that clinicians should not rely on the initial gap formation after the distalization of molars, misleadingly indicating success. On the contrary, the ongoing risk of anchorage loss throughout treatment should be considered. To achieve effective distalization distances exceeding 1 mm, employing additional appliances like sliders as temporary anchorage devices to fortify anchorage is recommended based on our findings. Moreover, our results underscore the necessity for further research employing meticulously designed studies on CAT to unravel existing system-related challenges and explore their underlying causes. Based on this, the predictability of translational mesio-distal and vertical movements can be improved in future orthodontic practice, thereby expanding the scope of CAT in orthodontic practice.

## Figures and Tables

**Table 1:** Distribution of distalization groups. Indication of teeth according to their planned distalization in mm.

Distalization Group	Distalization = Distal Translation	All Teeth	Incisors and Canines	Premolars	Molars
D1—slight distalization	0–1 mm	98	52	15	31
D2—moderate distalization	1.1–2 mm	111	43	39	29
D3—pronounced distalization	>2 mm	59	36	16	7

## Data Availability

The data presented in this study are available on request from the corresponding author.
